# Acceptability of Medical Male Circumcision Among Uncircumcised Men in Kenya One Year After the Launch of the National Male Circumcision Program

**DOI:** 10.1371/journal.pone.0019814

**Published:** 2011-05-16

**Authors:** Amy Herman-Roloff, Nixon Otieno, Kawango Agot, Jeckoniah Ndinya-Achola, Robert C. Bailey

**Affiliations:** 1 Division of Epidemiology and Biostatistics, University of Illinois at Chicago, Chicago, Illinois, United States of America; 2 Nyanza Reproductive Health Society, Kisumu, Kenya; 3 Impact Research and Development Organization, Kisumu, Kenya; 4 Department of Medical Microbiology, University of Nairobi, Nairobi, Kenya; Mayo Clinic, United States of America

## Abstract

**Background:**

Numerous studies have demonstrated that male circumcision (MC) reduces the incidence of the Type-1 human immunodeficiency virus (HIV) among heterosexual men by at least half.

**Methods:**

One year after the launch of a national Voluntary Medical Male Circumcision program in Kenya, this study conducted 12 focus group discussions among uncircumcised men in Nyanza Province to assess the revealed, non-hypothetical, facilitators and barriers to the uptake of MC.

**Results:**

The primary barriers to MC uptake included time away from work; culture and religion; possible adverse events; and the post-surgical abstinence period. The primary facilitators of MC uptake included hygiene; social pressure; protection against HIV and other sexually transmitted infections; and improved sexual performance and satisfaction.

**Conclusions:**

Some activities which might increase MC uptake include dispelling MC misconceptions; increasing involvement of religious leaders, women's groups, and peer mobilizers for MC promotion; and increasing the relevance of MC among men who are already practicing an HIV prevention method.

## Introduction

Male circumcision (MC) is the surgical removal of the foreskin of the penis and is practiced around the world for medical, religious, and cultural reasons. Over 40 observational studies and three randomized controlled trials (RCTs) have established that MC reduces the risk of the Human Immunodeficiency Virus (HIV) Type-1 acquisition in heterosexual men by approximately 60%.[1]–[4] In 2007, the World Health Organization (WHO) and the Joint United Nations Programme on HIV/AIDS (UNAIDS) recommended that MC, provided by trained professionals, be implemented as one component of a comprehensive HIV prevention strategy in regions with low MC rates, high HIV prevalence, and where heterosexual sex is the mode of transmission.[5]

Nyanza Province is the geographic home to the Luo ethnic group, a Niolotic-speaking people with some traditions and customs that differ from the surrounding primarily Bantu-speaking ethnic groups. The Luo people comprise the fourth largest ethnic group in Kenya with a population of approximately four million people.[6] A recent population survey reported that 21.5% of Luo men are circumcised and 17.1% are HIV-positive.[7] The Government of Kenya (GoK) launched the national Voluntary Medical Male Circumcision (VMMC) program in November, 2008, and plans to circumcise 860,000 males by 2013.[8] Currently, the VMMC program provides high-quality, medical MC services throughout Luo districts in Nyanza Province at no cost to clients.

Prior to completion of the three RCTs, several studies investigated factors that might facilitate or inhibit uptake of MC. Westercamp and Bailey reviewed 13 MC acceptability studies and concluded that the studies were consistent in identifying certain factors that facilitated MC uptake, including the beliefs that MC leads to improved hygiene, protection from sexually transmitted infections (STIs) and HIV, improved sexual pleasure and performance, and greater acceptability by other ethnic groups. The barriers to uptake most commonly identified were pain, culture and religion, cost, possible adverse events (AEs), and the potential for risk compensation (i.e., an increase in risky sexual behavior following MC).[9]

Studies conducted in Nyanza Province reported that the primary reasons men chose circumcision were enhanced protection from HIV and STIs, improved hygiene, decreased risk of penile cancer, and improved sexual satisfaction for men and their sex partners; while the primary reasons that men chose not to be circumcised were pain during/after the procedure, long healing period, AEs, culture or religion, and time away from work.[10]–[13] Reiss et al. reported that recently circumcised men said they were able to perform more rounds of sex; they were able to use condoms more easily; and they sustained fewer cuts on their penis during sex.[14]

Because of the consistency of the results of MC acceptability studies across several regions, as Westercamp and Bailey concluded, “…additional acceptability studies that pose hypothetical questions to participants are unnecessary.”[9] This study, conducted among uncircumcised men in Nyanza Province, Kenya, assessed the non-hypothetical barriers and facilitators of MC uptake after it was proven to be effective by the RCTs, was endorsed by the WHO and UNAIDS, was widely available at no cost, and was actively promoted by the GoK and implementing partners.

## Methods

### Ethics Statement

All research staff completed the online Collaborative Institutional Training Initiative training course on human subject protection. Written consent was obtained from all study participants. The study was approved by the University of Illinois at Chicago Institutional Review Board in Chicago, Illinois, USA (protocol: 2007-0913), and the Kenyatta National Hospital Ethics and Research Committee, in Nairobi, Kenya (protocol: P338/11/2007).

### Study Design

We conducted 12 focus group discussions (FGDs) in three out of the eight Luo districts within Nyanza Province. These three districts were chosen because they had an active VMMC program, were contiguous, and represented typical urban (Kisumu East) and rural (Nyando and Kisumu West) populations in Nyanza Province. FGDs were conducted between November-December, 2009, exactly one year after the launch of the VMMC program. The FGDs were moderated by one experienced male facilitator, and a research assistant took notes. The FGD guide consisted of 12 open-ended questions with probes about MC uptake and acceptability (Text S1) that were originally drafted in English, then translated into Kiswahili and Dholuo, and then verified by the research team. Established moderation techniques were employed to ensure active participation by all participants.[15]

Participants were recruited using a purposive sampling method at markets, shopping centers, and work places [16]; 121 men participated in the study. Because the aim was to explore the complete range of community opinions about MC among males most at risk for HIV acquisition, participants were recruited from urban and rural areas and from a variety of employment cadres common to the study area, including bicycle transporters (n = 32), students (n = 18), informal sector (n = 18), farmers (n = 12), shop/kiosk owners (n = 11), and other cadres, including teachers, fishermen, drivers, and religious leaders. To be eligible, potential participants had to be aged 18-40 years, be uncircumcised (based on self-report), have no plans to become circumcised, and reside in one of the three study districts where VMMC services were being widely provided.

Interested males were asked to participate in an informed consent process in the language of their choice (English, Dholuo, or Kiswahili), and if they chose subsequently to enroll, they provided signed consent. All enrolled males participated in the discussion, and no one terminated his participation prematurely. Each FGD lasted 60–90 minutes and involved 8–12 uncircumcised men. Six FGDs were conducted among “young men”, aged 18–27 years, and six FGDs were conducted among “older men” aged 28–40 years. For their time, participants were offered a snack, soda, and compensation of 200 Kenya Shillings ($2.50 USD), less than the average daily wage in Kenya.

### Data Collection and Management

Audio recordings were transcribed in the original language of the FGD, translated into English (if necessary), and then the translation was verified by a second staff member who compared it to the original transcript. The translated transcripts were reviewed, themes were identified, and a codebook was developed collaboratively by three members of the research team. All of the transcripts were imported into ATLAS.ti (version 6) and were coded independently by two research staff members; any discrepancies were discussed and a consensus was reached.

## Results

### Facilitators of male circumcision uptake

Three questions were asked to begin the discussion about factors that might act as facilitators to the uptake of MC services:

1. “What are some things people do to protect themselves, or their sexual partner, against getting HIV?”
2. “When you hear people talk about male circumcision in the community, what are some of the things they say?”
3. “A Luo man, named Onyango, is considering getting circumcised. What are some of the reasons that he might decide to get circumcised?”

(Please note, the character Onyango, a common name for a Luo man, was used throughout the FGD dialogue.)

The primary facilitators of MC uptake that were expressed in every discussion included the beliefs that MC improves hygiene, is influenced by social pressure, improves HIV and STI protection, and improves sexual performance and satisfaction, in order of salience.

#### Hygiene

Improved hygiene was the most common facilitator of MC. Participants described the improvement in hygiene resulting from MC in several ways, including: “good smelling”, “easy to wipe clean”, “no smell after sex or bathing”, and “HIV and other germs don't have a place to hide”.

#### Social pressure

Social pressure was a very common facilitator of MC uptake, especially among young men. Participants discussed, without prompting, several scenarios or social mechanisms that might affect Onyango's MC decision-making process.

1. A recently circumcised man shares his experience with Onyango and encourages him to become circumcised.
2. Onyango is the only one among his male friends or family who is not circumcised, and he is being teased about being uncircumcised, especially while bathing.
3. Onyango's female sex partner says that she will leave Onyango or withhold sex until he is circumcised.
4. Female sex partners and/or men from other ethnic groups might call Onyango “kehe”, or other derogatory names, to mean that he is a child and not a man since he is not circumcised.

Many participants remarked that if a man chose to be circumcised, he might be able to mix more freely with women and men from non-Luo ethnic groups in political, professional, and personal settings.

#### HIV and STI prevention

When participants were asked to discuss all HIV prevention methods, the “ABC” approach was the most common response, although other prevention strategies, such as HIV testing, were mentioned. One-third (2/6) of FGDs with young participants and 5/6 FGDs with older participants identified MC as an HIV prevention strategy without prompting. Additionally, when asked why Onyango might decide to get circumcised, HIV prevention was one of the reasons mentioned, although not the most common. A range of protective effect estimates were mentioned by the participants (range: 30%–100%). Most participants reported that they had heard that MC “reduces the chance of getting HIV”; however, they were confused or uncertain about two issues: 1) how MC protects against HIV acquisition; and 2) whether the MC-HIV connection is a myth or truly protective.

Although young men were less likely to identify MC as an HIV prevention strategy without prompting, they had more knowledge than older men about the HIV-MC association, and the mechanisms by which MC is protective against HIV. Some mechanisms discussed by young participants included MC “hardens the tip”, germs cannot live on a circumcised penis, and the foreskin has many HIV target cells.

Among older men, many comments about the MC-HIV association began with “Some say…,” indicating that they might have some skepticism or inadequate information about this association.

*Participant (P): “Some say that after circumcision you cannot acquire HIV easily.”*

*Moderator (M): “Why?”*

*P: “I don't know the reason but I hear them say so.”*

Participants also reported that MC reduced the incidence of STIs, specifically gonorrhea, syphilis, boils, and cervical cancer. The magnitude of the protective effect afforded by MC against HIV and STIs was often discussed as equivalent.

#### Sexual performance and satisfaction

Improved sexual performance and satisfaction, defined as male sexual satisfaction, female sexual satisfaction, and male sexual performance, were common facilitators to MC uptake, especially among young men. There was general consensus across all groups that MC improves sexual performance and satisfaction for men, and sexual satisfaction for female partners. Some reported that MC acts as a “natural condom,” and for this reason circumcised men can enjoy sex “skin-on-skin” without needing a latex condom. Additionally, participants reported that MC reduces cuts and bruising on the foreskin during sexual intercourse (this was discussed more by older men). Finally, participants believed that MC improves male sexual satisfaction by several other mechanisms, especially by reducing the worry of acquiring HIV or a STI during sex, by making condom use easier, and by making the penis more “rough” which increases friction during sex. Many participants reported additional sexual benefits for men after MC: men can have sex several times in the same night; the time to ejaculation is increased; penetration is easier; and circumcised men have more “energy” for sex.

The positive effect of MC on female sexual satisfaction was mentioned during all discussions. Most participants believed that women find circumcised men more sexually satisfying than uncircumcised men. Also, some participants believed that MC might encourage faithfulness if the female partners of circumcised men are more sexually satisfied. Finally, easier penetration, increased time to ejaculation, and increased friction were believed to affect female sexual satisfaction positively.

*P: “What women will tell him is that in the past other tribes have been saying that this [MC] can help with this job, it penetrates well and it's sweeter than the one that has not been cut.”*

#### Other facilitators

Other motives for MC uptake included the following beliefs: that adolescence is the ideal time for MC (11–18 years); that MC clients would receive material incentives ranging from soda to a substantial monetary compensation; that the Christian religion approves of MC because Jesus was circumcised; that MC is offered in a medical setting; and that parents, elders, and celebrities support MC.

Participants agreed that MC is preferable for males before they reach age 18 years; no participant discussed a man over 30 years being circumcised.

*P: “I've heard the old men say that circumcision should start at the age of 10 up to 18. Because beyond that, the muscles become mature.”*

Nearly all participants believed that MC services should be performed in a medical setting by a trained provider. Participants discussed several reasons for this opinion, but the two most common were these: 1) if MC is “medicalized,” it may minimize resistance among the Luo people because they will not see it as a counter-cultural practice being thrust on them; and 2) if MC is “medicalized,” AEs would be handled more propitiously by trained medical staff.

In September, 2008, before the launch of the national VMMC program in Kenya, Prime Minister Raila Odinga, a Luo political and cultural leader, encouraged Luo men to go for MC for HIV prevention. This endorsement especially impacted younger men.

*P: “As youths we go for circumcision because we are his disciples. So we will say, ‘If Raila did it, why not us?’ So we follow our leader.”*

### Barriers to male circumcision uptake

We asked participants, “What are some of the reasons that Onyango might decide not to get circumcised?” All groups responded that the primary barriers to MC uptake included (in order of salience): too much time away from work; cultural and religious values; the possibility of AEs; the post-surgical abstinence period; a desire to maintain the status quo; and increased promiscuity.

#### Time away from work

Participants reported that too much time away from work, especially if the man is the sole provider for the family, is the most significant barrier to seeking the service. This barrier was especially noted among older men, and men working in the informal sector, including bicycle transporters, security guards, fishermen and others. Participants believed that men might be away from work for a minimum of one week up to a maximum of 12 weeks after circumcision.

#### Cultural and religious values

Traditionally, the Luo removed the lower six teeth as a rite of passage into adulthood (while the neighboring Bantu groups practiced MC as a rite of passage). Recently, the practice of removing teeth has nearly ceased, and no practice has taken its place.[10] In this cultural context, the community has considered the role of MC as a medical practice versus a cultural rite.

Young participants viewed MC as a medical intervention that exists outside of culture, but older men often talked about MC as a cultural practice that is meant for other ethnic groups. Although young men discussed the health benefits afforded by MC, many still believed that getting the approval of elder males in their family was essential if one wished to be circumcised, and the consequences resulting from an unapproved circumcision could include being estranged from family, being forced to move off family land, and even dying.

*P: “In our community they [elders] say that circumcision is not good… They even say that that is the reason why the young people are dying because they are going against the rules of our ancestors.”*

Several participants, especially those who were young, reported that they believed that it would be a sin to get circumcised since circumcision would change God's creation. Older men talked about religion in the sense that if a man is “saved,” then he will not be promiscuous, and as a consequence, he will have no need for MC to protect him against HIV.

#### Adverse events

The possible incidence of AEs was a common barrier to uptake. The most common AEs discussed included pain and bleeding during- and post-MC, and delayed healing. Other AEs mentioned included negative effects on male reproduction resulting from the anesthetic injection, problems with appearance, torsion, infection, reduction in penile size, and surgical “accidents” that would mar appearance or impair function. Some participants noted that clients who have a bad experience will share their experience in the community.

*P: “So they [recently circumcised men] say there is a lot of bleeding… Another thing is some misconceptions like this local injection can cause you to be infertile in future and another thing is that an accident can occur…”*

#### Abstinence period

Almost all participants knew that an abstinence period of some duration was recommended after MC, and they discussed this as a barrier for both men and their female sex partners. Participants believed that men, especially young men, would be concerned that their female sex partners might seek other lovers while they are recovering. Older men reported that sleeping in the same bed with a wife would make it difficult to observe the abstinence period. Various durations of the abstinence period were discussed (range: 1 week – 8 weeks); some participants who knew the recommended duration of the abstinence period reported that six weeks was too long to abstain from sexual intercourse.

#### Status quo

Some participants believed that men did not need or desire MC. Several reasons were given to explain why MC is not “necessary”: the protection against HIV and STIs is not 100%, and if a man is already HIV-positive, has good hygiene, or is already practicing other HIV prevention methods (ABC method, HIV counseling and testing, etc.), he will not benefit from the procedure.

Participants offered other reasons, too, that men might not have the desire to go for MC: they and their sex partner(s) are already sexually satisfied; they do not wish to change the appearance or sensation of the penis; they are too old; and/or they do not want to introduce doubt in their relationship by going for MC.

*P: “When I told my wife that I wanted to go for circumcision, she told me that I am not faithful to her and so I want to go for circumcision so that I don't get infected. Secondly she told me that she liked that thing the way it was and she didn't want me to change it.”*

#### Promiscuity

The fear that MC will make a man promiscuous was mentioned frequently. On the individual level, participants said that if a man wants to get circumcised, his female sex partner(s), neighbors, and/or friends might think that he is promiscuous. As a community, it is believed that MC might create a generation of men, especially young men, who think that they can have sex without any risk. Some participants feared that if MC led to more promiscuity, it might produce more HIV transmission in the community, not less.

#### Other barriers

Other barriers to MC uptake included: a long distance to the health facility; a decrease in male and female sexual satisfaction; and peer influence against MC. Distance as a barrier to uptake was discussed in terms of reaching the facility, getting home from the facility (especially if the MC client is believed to be weak and in pain), and seeking follow-up care.

Discussed mechanisms for decreased male and/or female sexual satisfaction included less natural lubrication on a circumcised penis and decreased male penile sensitivity.

*P: “Even…circumcised persons have problems when having sex. In fact, when one is erect there are some fluids that lubricate him and after circumcision that place dries up and you'll be harming the girl because it's like you are stepping on her with a sole.”*

Finally, other barriers to MC uptake included opposition from girlfriends, reports from MC clients who say they have had a bad experience, and resistance from community leaders who oppose MC.

### Female service providers

Many organizations and governments providing MC services have wondered how MC clients would respond to female service providers. To explore this issue, we asked participants, “If Onyango goes to a health facility for male circumcision and finds the following, how might he react:

1. Female staff providing counseling and education on circumcision?
2. Female staff performing the circumcision?
3. Female staff attending to clients during follow-up visits?”

Most groups started this conversation thread by talking about interactions with female providers in non-professional terms, usually with sexual overtones. For example:

*P: “Onyango might be happy if he finds that it is a woman who performs the circumcision because he will be sure that the other girls will know that he has been circumcised and he can play sex perfectly.”*

The most common barrier discussed about a female service provider was that she might make a MC client feel “shy”. Some participants believed that Onyango might have an erection when a female provider touches or inspects his penis, thereby creating an awkward situation for both the provider and the client. However, by the end of the discussion, most participants concluded that as long as the provider was a trained professional, and the client did not know her, MC services being provided by females would not be a problem. A few participants believed that a female provider might perform services better than a male provider because women are more “understanding” and “gentle” when providing services compared to men.

### Community response

To explore how men expect their community to react to recently circumcised men, we asked participants, “In the end, Onyango decided to get circumcised. What would his neighbors say about Onyango if they found out he was circumcised?”

The range of expected community responses was wide, and participants attributed the variance to differences in age, education, and level of MC knowledge in the community. Older and/or less educated community members were expected to shun Onyango for abandoning his cultural traditions, while young and/or more educated community members were expected to congratulate Onyango and to be curious about the procedure and his experience.

*P: “It will depend with the kind of the neighbors that Onyango has. If they have the knowledge about circumcision they will encourage him. But if they are not informed, they will laugh at him and even isolate him because he has gone against their customs.”*

### Demand creation

We asked participants, “The Government of Kenya now recommends male circumcision for HIV prevention. What would be some of the ways to sensitize men, like Onyango, about the benefits and risks of male circumcision?”

Several sensitization approaches were discussed by participants. The following methods were proposed: radio broadcasts (in the local language so that people of all ages can understand them); women's groups who can then mobilize their husbands, sex partners, and male children; church leaders who can then mobilize the members of their congregation and the community; peer educators (using them to promote MC would create job opportunities for recently circumcised youth), sports rallies, and school curricula/programs.

## Discussion

While over 13 published studies indicated that MC was *likely* to be an acceptable HIV prevention strategy,[9] few studies have been published about revealed, non-hypothetical, preferences among uncircumcised men. Now that MC services are widely available at no cost, it is important to learn from uncircumcised men about the factors that influence MC uptake.

To a large extent, our results are consistent with findings from studies conducted prior to the scale-up of MC;[9]–[11] however, some differences are notable. Previous studies explored the impact of cost on MC uptake, but MC services are being provided at no cost in Kenya. Additionally, one study in Malawi reported that free services were viewed as poor quality,[17] but no participant in our study mentioned this; conversely, some believed that males might be more likely to adopt MC because the service is free. While previous acceptability studies explored the impact of time away from work as part of the total “cost” of the procedure, in this study, time away from work was the most important barrier to MC acceptability, especially among men working in the informal sector (e.g., bicycle transporters) and older men.

The WHO/UNAIDS recommends a six-week abstinence period following MC.[5] Participants in this study were aware that there is a recommended period of abstinence following MC; however, there was confusion about the duration of the period for abstinence, time away from work, and complete healing. National communication campaigns and couple's counseling should clarify these periods to ensure realistic expectations for MC clients and their sex partners, and to promote wound healing among recently circumcised men.

As noted in previous studies,[9] there was consensus that MC services should be offered in a medical setting, not in traditional settings, because a medical setting is believed to be more safe, and AEs can be handled by medical professionals. In this study, participants believed that medical male circumcision also clarifies the purpose of the national VMMC program – that is, VMMC is not trying to change, or dilute, any ethnic group's culture; instead, it is promoting MC for medical and health purposes. This might be an important distinction to be made in other regions where MC is being promoted.

When asked about HIV prevention methods, participants were most familiar with the “ABC” approach and frequently did not situate MC within their existing HIV prevention framework – this was especially true among young men. This trend was observed in Uganda where older men were significantly more aware of MC for HIV prevention than youths, and overall, only 38.2% of respondents mentioned MC as an HIV prevention strategy in an open-ended question.[18] In our study 7/12 groups reported MC as an HIV prevention strategy without prompting. While older men were more aware of MC for HIV prevention, they were less likely to believe that MC was necessary, especially if a man practiced other HIV prevention methods. This disparity between knowledge and beliefs is important, and should be explored in program implementation and future research.

Several studies are on-going to assess the potential for risk compensation, or the increase in sexually risky behaviors, post-MC. Among the participants in this study, there was a wide range in estimates of the protective effect afforded by MC against HIV acquisition (range: 30%–100%). Similar to results reported by Reiss et al. and Wilcken et al., most participants did not know the exact magnitude of the protective effect,[14], [18] but they knew that MC was not fully protective and that other HIV prevention methods would continue to be necessary. However, some participants believed that men might be motivated to seek MC services because they want to have sex without a condom and/or increase their number of sexual partners. Based on these beliefs, risk compensation remains a possibility, especially when services are provided with less counseling and less-or-no recurrent contact with MC clients than during the RCTs. It remains important for a national communication strategy to continue enforcing the general knowledge that MC is only partially protective against HIV acquisition and to clarify the magnitude of the protective effect.

The possibility of discrimination was discussed by participants in this study; specifically, it was believed that some members of the community might shun recently circumcised men, especially when community members are older and/or less educated; this finding is consistent with findings from pervious studies.[9] While some participants in this study believed that men might seek MC services because Jesus was circumcised, others, especially young men, reported that it was a sin for men to change the way they were created. This ambiguity, and lack of consensus, has been observed in the literature.[9] Finally, Westercamp and Bailey recommended that it would be “prudent to consult and collaborate with religious leaders” before promoting MC in a country.[9] This remains an important recommendation in scaling-up MC services throughout Africa.

Circumcision for adult men (over 18 years) was a barrier to MC adoption expressed by many in this study, and this is consistent with the findings of previous studies. Studies have reported that circumcision at 7–13 years of age is most preferable because a boy at this level of maturity can make the decision for himself, understand the significance of the event, take care of the wound himself, and is unlikely to have initiated sexual activity.[10], [17] In this sample, participants believed that adolescent boys and teenagers were best suited to go for MC (aged 11–18 years). While several governments are targeting sexually active males during the initial phase of implementing a large-scale MC program,[5], [8] community opinions about the ideal age for MC should not be ignored.

The results of this study might be useful for the development or improvement of a MC communication campaign both within and outside of Kenya. A national or regional communication campaign is well-positioned to dispel misconceptions like the recommended duration of time away from work (usually a few days, but less than one week) versus the recommended abstinence period (six weeks). Additionally, many participants stated that men are concerned about developing severe AEs post-MC. Preliminary results from a study in Kenya found that 2.7% of clients experienced a moderate or severe AE, and all AEs resolved with treatment;[12] low AE rates were also reported by a large-scale MC program in South Africa.[19] The message that medical MC very rarely results in severe, untreatable AEs should be disseminated widely. Finally, participants were hesitant to believe that MC really protects against HIV acquisition because they did not know the mechanisms that explained this protection, and these mechanisms can be communicated through campaign messages.

The findings reported above should be considered along with the following study limitations. The results from this study might not be generalizable to other programs and countries since they were restricted to Nyanza Province. Additionally, while purposive sampling was employed in an attempt to recruit a representative sample of uncircumcised men (self-report) aged 18–40 years, it is possible that this study might not describe the full range of beliefs related to MC in Nyanza Province; however, saturation was achieved, and no new themes emerged during the final FGDs conducted within each age category. Also, all participants were uncircumcised with no plans to get circumcised, so their opinions about MC might be more negative than the general population. Finally, the data collected during this study were self-reported opinions about community perceptions of MC; therefore, it is possible that the participants themselves did not hold these opinions and might have withheld, or exaggerated, information.

The results of this study are very consistent with the results of previous studies, but they add a nuanced understanding of revealed – not hypothetical – acceptability of MC services. These results may be used to implement or improve several program activities to positively impact MC uptake, including: revising communication messages to dispel misconceptions; increasing the involvement of religious leaders, women's groups, and peer mobilizers in MC sensitization; and situating MC within the existing HIV prevention framework (e.g., ABC, HIV testing, home-based counseling and testing, couples testing and counseling, and STI diagnosis and treatment.) to improve the relevance of this intervention for men already practicing some HIV prevention methods.

## Supporting Information

Text S1 Focus Group Discussion Guide.
(DOC)
