# Supplementary material for: Acceptability of Medical Male Circumcision Among Uncircumcised Men in Kenya One Year After the Launch of the National Male Circumcision Program
Source: PLoS One. 2011 May 16;6(5):e19814. doi: 10.1371/journal.pone.0019814 (PMC3095626; doi:10.1371/journal.pone.0019814)
Supplement: Text S1 — Focus Group Discussion Guide. (DOC) [file pone.0019814.s001.doc]

**Focus Group Discussion Guide**

## HIV Context

1. What are some of the words or phrases that people in this community use when talking about HIV/AIDS?
   1. *Let’s go around the room, and share one or two words or phrases that come to mind. (If coded words/phrases are used, please probe for their meaning.)*
2. What are some things people do to protect themselves, or their sexual partner, against getting HIV?

Male Circumcision Acceptability

1. When you hear people talk about male circumcision in the community, what are some of the things they say?
   1. *What are some of the ways male circumcision is described?*
   2. *What are some of the things that you have heard discussed about the relationship between male circumcision and HIV?*
2. Imagine that two young Luo men are having a conversation about male circumcision. What are some of the things they might say?
   1. *What are some of the reasons they are discussing circumcision?*
   2. *What are some of the things that they would say about male circumcision in the context of Luo culture?*
   3. *What are some of the things that they would say about circumcision and a man’s health?*
   4. *What other things might they be discussing about circumcision?*
3. Imagine that two Luo elders are having a conversation about male circumcision. What are some of the things they might say?
   1. *What are some of the ways that their conversation might differ from the conversation between young, Luo men?*
4. A Luo man, named Onyango, is considering getting circumcised. What are some of the reasons that he might decide to get circumcised?
   1. *How would hygiene impact his decision?*
   2. *How would sexual pleasure impact his decision?*
   3. *How would protection from HIV impact his decision?*
   4. *How would protection from STIs impact his decision?*
   5. *Are there any other reasons?*
5. What are some of the reasons that Onyango might decide not to get circumcised?
   1. *How would cost impact his decision?*
   2. *How would travel to and from the health facility impact his decision y?*
   3. *How would abstinence from sex impact his decision?*
   4. *How would pain impact his decision?*
   5. *How would time off from work impact his decision?*
   6. *How would infection impact his decision?*
   7. *How would the Luo culture impact his decision?*
   8. *How would stigma impact his decision?*
   9. *Are there any other reasons?*
6. If Onyango goes to a health facility for male circumcision and finds the following, how might he react:
   1. *Female staff providing counseling and education on circumcision?*
   2. *Female staff assisting in the theater with the circumcision?*
   3. *Female staff performing the circumcision?*
   4. *Female staff attending to clients during follow-up visits?*
7. To help him in his decision, Onyango decides to talk with his female sex partners about him getting circumcised. What are some of the things that these women might say?
   1. *What are some of the things that the women might say to convince Onyango to get circumcised?*
   2. *What are some of the things that the women might say to convince Onyango not to get circumcised?*
   3. *What are some of the ways that women should be involved in the decision of their partners to become circumcised?*
8. The Government of Kenya now recommends male circumcision for HIV prevention. What would be some of the ways to sensitize men, like Onyango, about the benefits and risks of male circumcision?
   1. *What are some of the groups that should be involved in sensitizing men about male circumcision?*
9. In the end, Onyango decided to get circumcised. What would his neighbors say about Onyango if they found out he was circumcised?
   1. *What are some of the positive things they might say?*
   2. *What are some of the negative things they might say?*

## Summary Question

1. Are there any other factors pertaining to male circumcision that we missed, but should discuss?
